# Fatal tuberculous brain abscesses in an immunocompetent woman

**DOI:** 10.11604/pamj.2015.20.372.6404

**Published:** 2015-04-15

**Authors:** Hassen Ben Ghezala, Salah Snouda

**Affiliations:** 1 Service Universitaire des Urgences et de Réanimation Médicale, Faculté de Médecine de Tunis, Hôpital Régional de Zaghouan, Tunisie

**Keywords:** Tuberculosis, encepahlitis, immunocompetent, intensive care unit

## Image in medicine

**Figure 1:**
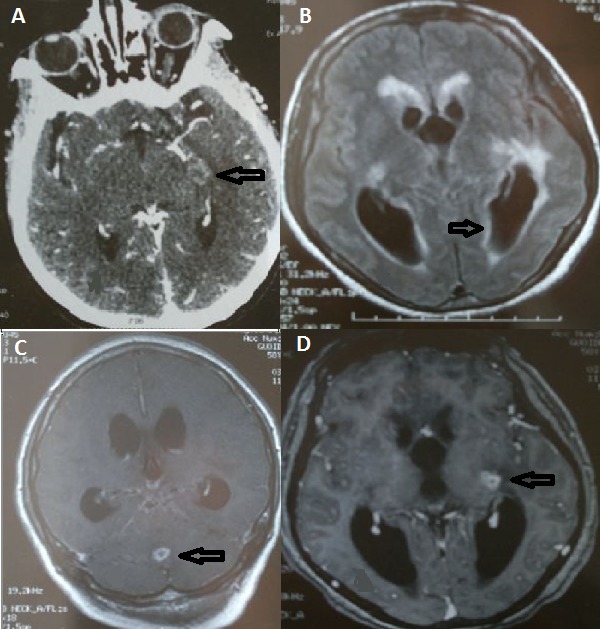
Brain imaging signs of tuberculous meningoenpahlitis: CT scan and MRI: A) temporal abscess in brain CT scan after injection of contrast; B) dilated ventricles (hydrocephalus) in magnetic resonance imaging (MRI); C) Vvrmian abscess in MRI T1 weighted; D) temporal abscess in MRI T1 weighted

The identification of the cause of acute encephalitis is difficult in spite of many developed diagnosis strategies. The cause is unknown in nearly 30 to 40% of cases. Tuberculosis is endemic in Tunisia. Tuberculous encephalitis is rare in immunocompetent adults. We report in this work a rare case report of tuberculous encephalitis in an immunocompetent woman. She was 38 years old and had no medical history. She had a clinical presentation of mental confusion and fever that appeared from ten days. She was agitated with neck stiffness. Her initial Glasgow coma scale was thirteen. The CT scan of the head showed a brain temporal abscess (A). The cerebral spinal fluid examination showed elevated proteinorrachia of 172 mg/dl, normal glycorrachia and lymphocytic pleocytosis (76% of 340 WBC/mm3). The Magnetic resonance imaging (MRI) of the head performed in the second day revealed images of hydrocephalus (B). It showed also two images of brain vermian (C) and temporal abscesses (D). The diagnosis of tuberculous encephalitis was made by computing clinical, laboratory and imaging findings. She was admitted in the intensive care unit and treatment by cefotaxim and acyclovir was initiated. Antituberculous drugs were added at the second day of management after MRI. In the sixth day of management, the patient became comatose and required intubation and mechanical ventilation. She died three days later from neurologic complications. Figure 1

